# The association between hospital length of stay before rapid response system activation and clinical outcomes: a retrospective multicenter cohort study

**DOI:** 10.1186/s12931-021-01660-9

**Published:** 2021-02-18

**Authors:** Jimyung Park, Yeon Joo Lee, Sang-Bum Hong, Kyeongman Jeon, Jae Young Moon, Jung Soo Kim, Byung Ju Kang, Jong-Joon Ahn, Dong-Hyun Lee, Jisoo Park, Jae Hwa Cho, Sang-Min Lee

**Affiliations:** 1 Division of Pulmonary and Critical Care Medicine, Department of Internal Medicine, Seoul National University College of Medicine, 101, Daehak-ro, Jongno-gu, Seoul, 03080 Republic of Korea; 2 Division of Pulmonary and Critical Care Medicine, Department of Internal Medicine, Seoul National University Bundang Hospital, Seongnam-si, Republic of Korea; 3 Department of Pulmonary and Critical Care Medicine, Asan Medical Center, University of Ulsan College of Medicine, Seoul, Republic of Korea; 4 Department of Critical Care Medicine, Samsung Medical Center, Sungkyunkwan University School of Medicine, Seoul, Republic of Korea; 5 Division of Pulmonary and Critical Care Medicine, Department of Medicine, Samsung Medical Center, Sungkyunkwan University School of Medicine, Seoul, Republic of Korea; 6 Division of Pulmonary and Critical Care Medicine, Department of Internal Medicine, Chungnam National University Sejong Hospital, Chungnam National University College of Medicine, Sejong-si, Republic of Korea; 7 Division of Pulmonary and Critical Care Medicine, Department of Internal Medicine, Inha University Hospital, Inha University School of Medicine, Incheon, Republic of Korea; 8 Department of Internal Medicine, Ulsan University Hospital, University of Ulsan College of Medicine, Ulsan, Korea; 9 Division of Pulmonary and Critical Care Medicine, Department of Internal Medicine, Dong-A University College of Medicine, Busan, Korea; 10 Division of Pulmonology, Department of Internal Medicine, CHA Bundang Medical Center, CHA University, Seongnam-si, Korea; 11 Division of Pulmonology, Department of Internal Medicine, Gangnam Severance Hospital, Yonsei University College of Medicine, Seoul, Korea

**Keywords:** Hospital rapid response team, Clinical deterioration, Length of stay, Mortality, General ward, Intensive care units

## Abstract

**Background:**

Rapid response system (RRS) is being increasingly adopted to improve patient safety in hospitals worldwide. However, predictors of survival outcome after RRS activation because of unexpected clinical deterioration are not well defined. We investigated whether hospital length of stay (LOS) before RRS activation can predict the clinical outcomes.

**Methods:**

Using a nationwide multicenter RRS database, we identified patients for whom RRS was activated during hospitalization at 9 tertiary referral hospitals in South Korea between January 1, 2016, and December 31, 2017. All information on patient characteristics, RRS activation, and clinical outcomes were retrospectively collected by reviewing patient medical records at each center. Patients were categorized into two groups according to their hospital LOS before RRS activation: early deterioration (LOS < 5 days) and late deterioration (LOS ≥ 5 days). The primary outcome was 28-day mortality and multivariable logistic regression was used to compare the two groups. In addition, propensity score-matched analysis was used to minimize the effects of confounding factors.

**Results:**

Among 11,612 patients, 5779 and 5883 patients belonged to the early and late deterioration groups, respectively. Patients in the late deterioration group were more likely to have malignant disease and to be more severely ill at the time of RRS activation. After adjusting for confounding factors, the late deterioration group had higher 28-day mortality (aOR 1.60, 95% CI 1.44–1.77). Other clinical outcomes (in-hospital mortality and hospital LOS after RRS activation) were worse in the late deterioration group as well, and similar results were found in the propensity score-matched analysis (aOR for 28-day mortality 1.66, 95% CI 1.45–1.91).

**Conclusions:**

Patients who stayed longer in the hospital before RRS activation had worse clinical outcomes. During the RRS team review of patients, hospital LOS before RRS activation should be considered as a predictor of future outcome.

## Background

A rapid response system (RRS) is a system designed for prompt and appropriate intervention in patients who experience unexpected clinical deterioration during hospitalization [1]. Previous studies have shown its efficacy in reducing the rates of in-hospital mortality, and the implementation of RRS has been increasing worldwide in recent decades [2, 3]. Therefore, accurate risk stratification is needed for improving the triage of patients and resource allocation. However, despite the pervasive use of RRS, little is known about how best to predict the clinical outcomes of patients for whom RRS is activated. A recent study using multicenter registry data reported that, in addition to vital signs such as systolic blood pressure and respiratory rate, time after admission before RRS activation is a significant predictor of mortality [4].

Hospital length of stay (LOS) is often used as one method for assessing patient outcomes in clinical practice. It is well known that a longer hospital LOS is associated with a higher risk of malnutrition and frailty [5, 6]. In addition, older patients with poor baseline functional status are more likely to stay longer after various elective surgeries because of postoperative complications [7]. Considering that RRS activation occurs in the middle of hospitalization while patients are being actively treated, hospital LOS before RRS activation may provide a simple substitute marker for estimating the effectiveness of past treatments and predicting future outcomes. In this study, using a large nationwide multicenter database of patients reviewed by RRS teams, we aimed to evaluate whether hospital LOS before RRS activation can predict the clinical outcomes of patients after adjusting for confounding factors associated with the severity of illness.

## Methods

### Study population

We retrospectively analyzed the medical records of patients for whom an RRS call was activated in 9 tertiary referral hospitals in South Korea between January 1, 2016, and December 31, 2017. Patients older than 18 years for whom RRS was activated during hospitalization were eligible for this study and screened using the RRS database for each center. We included every eligible patient whose time after admission before RRS activation was ≤ 1 month. If there were recurrent RRS calls in one patient, the first circumstance of RRS activation was included in the analysis of this study. The study protocol was reviewed and approved by the institutional review board of each center and informed consent was waived.

### Implementation of RRS

Although the detailed protocols of RRS differed between centers, all centers used the vital signs or certain laboratory results for in-ward patients to screen and identify patients at risk of clinical deterioration. In addition, calls from medical staff members on the ward, including doctors and nurses, were also used to identify patients whose clinical situation was worsening, even if they did not meet the specific activation criteria, and the RRS team was dispatched if needed. The detailed RRS activation criteria are summarized in the Additional file 1: Appendix S1.

### Study outcomes and data collection

The primary outcome of our study was 28-day mortality. For secondary outcomes, we evaluated in-hospital mortality, admission to the intensive care unit (ICU), hospital LOS after RRS activation, and LOS in the ICU for patients admitted to the ICU. Data were collected retrospectively by reviewing patient medical records at each participating center. Detailed information regarding the situation when RRS was activated was collected. In addition to the interventions carried out by the RRS team, we also checked whether discussion of a do-not-resuscitate (DNR) order was made after RRS activation. The severity of illness at the time of RRS activation was evaluated, and the modified early weaning score (MEWS) and the national early warning score 2 (NEWS2) were calculated for each patient.

### Statistical analysis

To evaluate the effects of hospital LOS before RRS activation on the clinical outcomes, we categorized the patients according to their days since admission before RRS activation. We divided patients into two groups (early deterioration vs. late deterioration groups) by dichotomizing hospital LOS before RRS activation with the median value. Differences between groups were analyzed using the Mann–Whitney U test or Pearson’s chi-squared test, as appropriate. *P* values < 0.05 for two-tailed tests were considered to be statistically significant.

Mortality outcomes and rates of admission to the ICU were compared between the early and late deterioration groups using a multivariable logistic regression and adjusted odds ratios (aORs) with 95% confidence intervals (CIs) were calculated. We selected relevant clinical variables to adjust for that showed significant associations with mortality outcomes. Missing variables were handled with multiple imputation methods [8]. Outcomes related to LOS (hospital LOS after RRS activation and LOS in the ICU for patients admitted to the ICU) were analyzed using a negative binomial regression to calculate the adjusted incidence rate ratios (aIRRs).

Even after using multivariable regression models to compare clinical outcomes between the early and late deterioration groups, we expected some remaining confounding factors to be present because the two groups would be clinically different. Therefore, we performed propensity score-matched analysis to reduce the effects of possible confounding factors as much as possible [9]. After calculating propensity score (late vs. early deterioration), we conducted 1:1 optimal matching without replacement [10]. A more detailed description of the analysis is available in the Additional file 1: Appendix S2. Using matched samples, we calculated unadjusted and adjusted ORs to assess the associations between binary outcomes and LOS before RRS activation using a conditional logistic regression model for matched data [11, 12]. For continuous outcomes, the Wilcoxon signed-rank test was used to compare between matched samples.

We also performed several sensitivity analyses. First, we included the patients who were excluded in the main analysis because RRS was activated after 1 month of admission to evaluate whether our inclusion criteria affected the study results. Second, we conducted analysis with only patients without missing data to exclude possible bias due to the multiple imputation methods used to handle missing data. Third, patients in whom discussion on DNR orders occurred were excluded for the analysis. Fourth, the same analyses were repeated with handling hospital LOS as a continuous variable, instead of categorizing patients into early and late deterioration groups, to examine the robustness of our results. Fifth, we categorized patients into four quartile groups using the interquartile range (IQR) values for the hospital LOS before RRS activation. Then, we evaluated whether there was a proportional relationship between the LOS before RRS activation and mortality outcomes.

For the primary outcome (28-day mortality), prespecified subgroup analyses with tests for interactions were performed. We compared the effects of hospital LOS on the clinical outcomes between subgroups according to the patient’s department of admission (medical vs. surgical) and whether the patient had undergone a surgical operation before RRS activation. In addition, we analyzed the subgroup of patients for whom RRS was activated through use of the screening criteria (i.e. not by calls from in-ward medical staff members). We also conducted subgroup analyses for three key comorbidities (solid cancer, hematological malignancy, and chronic lung disease). All statistical analyses were performed using STATA software (version 14.0; StataCorp LP, College Station, TX, USA).

## Results

### Patient characteristics

**Fig. 1:**
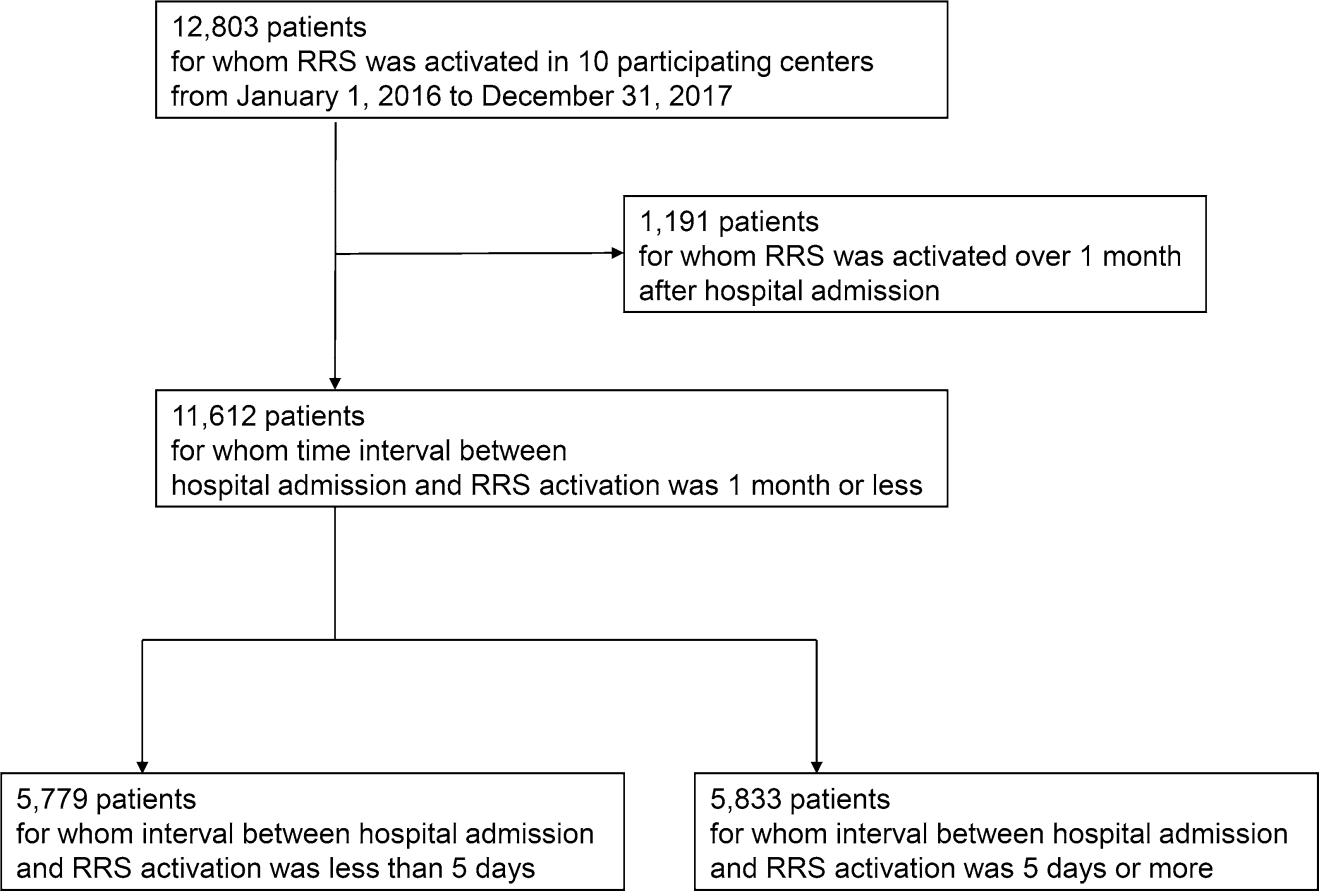
Flowcharts of patients included in the study

During the 2-year study period, a total of 12,803 patients had RRS activation in 9 participating centers. Of these patients, 1191 were excluded because RRS activation occurred > 1 month after admission to the hospital and 11,612 patients were included in our analysis (Fig. 1). The excluded patients were younger and had lower BMI. They were also more likely to be admitted for surgical operation or hematological malignancy (Additional file 1: Table S1). The median number of days after admission before RRS activation was 5 (IQR 2–10) (Additional file 1: Fig. S1). Using a cutoff value of 5 days, we divided patients into two groups: an early deterioration group (LOS before RRS activation < 5 days, N = 5779) and a late deterioration group (LOS before activation ≥ 5 days, N = 5883).

The baseline characteristics were compared between these two groups and are summarized in Table 1. Patients in the early deterioration group were more likely to be admitted to a medical department than a surgical department, and significantly more patients in the late deterioration group underwent a surgical operation before RRS activation during their hospitalization. The comorbidities differed between the two groups. Particularly, malignant disease was more common in the late deterioration group, whereas chronic lung disease was more frequent in the early deterioration group.

**Table 1:** Patient characteristics

Variables	Total	Early	Late	*P* value
	patients	deterioration	deterioration	
	(N = 11,612)	(N = 5779)	(N = 5833)	
Age (year)	68 (57–76)	68 (57–77)	67 (57–76)	0.288
Sex				
Male	6785 (58.4%)	3265 (56.5%)	3520 (60.4%)	< 0.001
Female	4827 (41.6%)	2514 (43.5%)	2313 (39.6%)	
Body mass index (kg/m^2^)	22.2 (19.6–25.0)	22.3 (19.8–25.0)	22.2 (19.5–24.9)	0.012
Admission department				
Medical	8160 (70.3%)	4120 (71.3%)	4040 (69.3%)	0.017
Surgical and obstetrical	3452 (29.7%)	1659 (28.7%)	1793 (30.7%)	
Surgical operation	2867 (24.7%)	1050 (18.2%)	1817 (31.2%)	< 0.001
Before RRS activation				
Postoperative days^a^ (days)	3 (1–7)	1 (0–2)	5 (3–10)	< 0.001
Comorbidity				
Solid cancer	4646 (40.0%)	2238 (38.7%)	2408 (41.3%)	0.005
Hematological malignancy	1002 (8.6%)	336 (5.8%)	666 (11.4%)	< 0.001
Cardiovascular disease	2715 (23.4%)	1323 (22.9%)	1392 (23.9%)	0.216
Diabetes mellitus	3132 (27.0%)	1520 (26.3%)	1612 (27.6%)	0.105
Chronic lung disease	1687 (14.5%)	909 (15.7%)	778 (13.3%)	< 0.001
Hepatobiliary disease	1297 (11.2%)	640 (11.1%)	657 (11.3%)	0.747
Chronic kidney disease	1251 (10.8%)	590 (10.2%)	661 (11.3%)	0.051
Cerebrovascular disease	1329 (11.5%)	585 (10.1%)	744 (12.8%)	< 0.001
Organ transplantation	356 (3.1%)	128 (2.2%)	228 (3.9%)	< 0.001
Hospital LOS before	5 (2–10)	2 (1–3)	10 (7–17)	< 0.001
RRS activation (days)				

*RRS* rapid response system, *LOS* length of stay

^a^Postoperative days are summarized for only patients who were admitted and received a surgical operation

### RRS activations

Information about the context of RRS is described in Additional file 1: Table S2. The most common assessment made by the RRS team was respiratory distress (35.1%). At the time of RRS activation, more severely ill patients were included in the late deterioration group. Hence, interventions delivered by the RRS team also differed between the two groups (Table 2). Notably, discussion on DNR orders occurred significantly more frequently in the late deterioration group.

**Table 2:** Severity of illness at RRS activation and interventions by RRS team

Variables	Total	Early	Late	*P* value
	patients	deterioration	deterioration	
	(N = 11,612)	(N = 5779)	(N = 5833)	
Mean blood pressure (mmHg)	83 (66–98)	82 (66–97)	83 (66–98)	0.289
Heart rate (rate/min)	102 (84–120)	100 (82–120)	104 (86–122)	< 0.001
Respiratory rate (rate/min)	22 (18–28)	22 (18–28)	22 (18–28)	0.46
Body temperature (°C)	36.9 (36.5–37.6)	36.9 (36.5–37.6)	36.9 (36.5–37.6)	0.36
Alert mental status	7968 (68.6%)	4204 (72.8%)	3764 (64.5%)	< 0.001
Need for supplementary oxygen therapy	6880 (59.3%)	3339 (57.8%)	3541 (60.7%)	0.001
MEWS	4 (2–5)	4 (2–5)	4 (2–6)	< 0.001
NEWS2	7 (5–10)	7 (5–10)	8 (5–10)	< 0.001
SOFA score^a^	5 (3–7)	4 (2–7)	5 (3–8)	< 0.001
Intervention of RRS team				
Tracheal intubation	1525 (13.1%)	720 (12.5%)	805 (13.8%)	0.032
Mechanical ventilation	1400 (12.1%)	655 (11.3%)	745 (12.8%)	0.017
High flow nasal cannula	900 (7.8%)	458 (7.9%)	442 (7.6%)	0.484
Noninvasive ventilation	172 (1.5%)	103 (1.8%)	69 (1.2%)	0.008
Renal replacement therapy	499 (4.3%)	241 (4.2%)	258 (4.4%)	0.501
ACLS including CPR	453 (3.9%)	201 (3.5%)	252 (4.3%)	0.019
ECLS	49 (0.4%)	26 (0.5%)	23 (0.4%)	0.644
Central venous catheter	831 (7.2%)	415 (7.2%)	416 (7.1%)	0.918
Antimicrobial therapy	745 (6.4%)	325 (5.6%)	420 (7.2%)	0.001
Vasopressor/inotrope	1443 (12.4%)	705 (12.2%)	738 (12.7%)	0.46
Transfusion	659 (5.7%)	307 (5.3%)	352 (6.0%)	0.093
Discussion regarding	1226 (10.6%)	541 (9.4%)	685 (11.7%)	< 0.001
do-not-resuscitate order				

*RRS* rapid response system, *MEWS* modified early weaning score, *NEWS2* national early warning score 2, *SOFA* sequential organ failure assessment, *ACLS* advanced cardiac life support, *CPR* cardiopulmonary resuscitation, *ECLS* extracorporeal life support

^a^SOFA scores were available in 6,733 (58.0%) patients who had relevant laboratory test results including arterial blood gas analysis

### Outcomes in relation to the hospital LOS before RRS activation

Data on primary and secondary outcomes are summarized in Table 3. Overall, 2534 of 11,612 patients (21.8%) had died by 28 days after RRS activation: 1051 of 5779 patients (18.2%) in the early deterioration group and 1483 of 5833 patients (25.4%) in the late deterioration group (aOR 1.60; 95% CI 1.44–1.77). The effects of other adjusted variables are described in Additional file 1: Table S3. Similar results were observed in the analysis of in-hospital mortality with a higher mortality rate in the late deterioration group (20.9% vs. 30.2%, aOR 1.71; 95% CI 1.55–1.88). Other clinical outcomes (ICU admission, hospital LOS after RRS activation, and LOS in the ICU) were all worse in the late deterioration group.

**Table 3:** Effects of the hospital LOS before RRS activation on the clinical outcomes

Variables	Early deterioration (N = 5779)	Late deterioration (N = 5833)	*P* value
Primary outcome			
28-day mortality	1051 (18.2%)	1483 (25.4%)	
Unadjusted OR	1.00	1.53 (1.40–1.68)	< 0.001
Adjusted OR^1^	1.00	1.60 (1.44–1.77)	< 0.001
Secondary outcome			
In-hospital mortality	1209 (20.9%)	1758 (30.2%)	
Unadjusted OR	1.00	1.63 (1.50–1.78)	< 0.001
Adjusted OR^a^	1.00	1.71 (1.55–1.88)	< 0.001
ICU admission	1611 (27.9%)	1774 (30.4%)	
Unadjusted OR	1.00	1.13 (1.04–1.23)	0.003
Adjusted OR^a^	1.00	1.10 (1.01–1.20)	0.030
Hospital LOS after RRS activation (days)	10 (5–20)	14 (6–27)	
Unadjusted IRR	1.00	1.33 (1.28–1.38)	< 0.001
Adjusted IRR^a^	1.00	1.30 (1.25–1.35)	< 0.001
LOS in ICU^b^ (days)	4 (2–9)	5 (3–10)	
Unadjusted IRR	1.00	1.21 (1.13–1.30)	< 0.001
Adjusted IRR^a^	1.00	1.22 (1.14–1.31)	< 0.001

*LOS* length of stay, *RRS* rapid response system, *OR* odds ratio, *IRR* incidence rate ratio, *ICU* intensive care unit

^a^Multivariable logistic regression and negative binomial regression were performed with adjusting for following confounding variables: age, department of admission (medical vs. surgical), whether patients were in postoperative state, comorbidities of solid cancer, hematological malignancy, chronic lung disease, hepatobiliary disease, or cerebrovascular disease, whether DNR (do-not-resuscitate) discussion was made after RRS activation, and NEWS2 (national early warning score 2)

^b^Analysis on LOS in ICU included only patients who were admitted to the ICU

### Propensity score-matched analysis

Among these 10,149 patients who had no missing data for the prespecified variables for the propensity model, 4454 patients were 1:1 matched for each of the early and late deterioration groups. The distribution of the propensity scores in the two groups is depicted in Additional file 1: Fig S2. The matched groups were shown to be well balanced in baseline characteristics (Additional file 1: Table S4), except that more patients in the late deterioration group underwent a surgical operation during hospitalization before RRS activation (standardized difference 11.1%).

The results of analysis of the propensity score-matched samples are summarized in Table 4. The associations between mortality outcomes and hospital LOS before RRS activation were similar to those found in the unmatched analysis. The late deterioration group had a significantly higher rates of 28-day mortality (aOR 1.66; 95% CI 1.45–1.91) and in-hospital mortality (aOR 1.72; 95% CI 1.51–1.95), which showed similar aOR values as in the unmatched analysis.

**Table 4:** Analysis in the propensity score-based 1:1 matched patients

Variables	Early deterioration (N = 4454)	Late deterioration (N = 4454)	*P* value^c^
Primary outcome			
28-day mortality	859 (19.3%)	1108 (24.9%)	
Unadjusted OR	1.00	1.39 (1.26–1.54)	< 0.001
Adjusted OR^1^	1.00	1.66 (1.45–1.91)	< 0.001
Secondary outcome			
In-hospital mortality	999 (22.5%)	1309 (29.4%)	
Unadjusted OR	1.00	1.45 (1.31–1.59)	< 0.001
Adjusted OR^a^	1.00	1.72 (1.51–1.95)	< 0.001
ICU admission	1325 (29.8%)	1362 (30.6%)	
Unadjusted OR	1.00	1.04 (0.95–1.14)	0.392
Adjusted OR^a^	1.00	1.10 (0.99–1.22)	0.080
Hospital LOS after RRS activation (days)	11 (5–21)	14 (6–27)	< 0.001
LOS in ICU^b^ (days)	4 (2–9)	5 (3–10)	0.305

*LOS* length of stay, *RRS* rapid response system, *OR* odds ratio, *IRR* incidence rate ratio, *ICU* intensive care unit

^a^To minimize effects of remaining confounding factors after matching, adjusted analyses were also performed. Following variables were adjusted: age, department of admission (medical vs. surgical), whether patients were in postoperative state, comorbidities of solid cancer, hematological malignancy, chronic lung disease, hepatobiliary disease, or cerebrovascular disease, whether DNR (do-not-resuscitate) discussion was made after RRS activation, and NEWS2 (national early warning score 2)

^b^Analysis on LOS in ICU included only patients who were admitted to the ICU

^c^*P* values were calculated by conditional logistic regression for binary outcomes and by Wilcoxon signed-rank test for continuous outcomes

### Sensitivity analysis

**Fig. 2:**
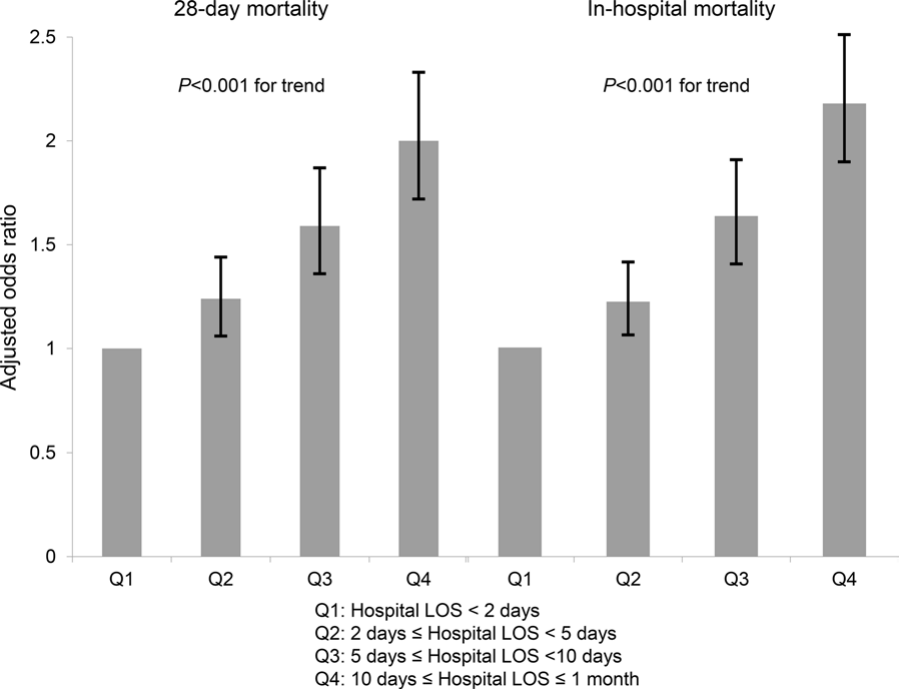
Adjusted odds ratio for 28-day mortality and in-hospital mortality according to quartile groups

Several sensitivity analyses showed the robustness of the results of our main analysis (Additional file 1: Table S5–9). Particularly, when hospital LOS was handled as a continuous variable in the regression analysis, 1-day increase in hospital LOS was associated with worsening of all clinical outcomes, consistent with the results of main analysis. Then, we performed further analysis after dividing patients into four quartiles (Qs) according to their hospital LOS before RRS activation: Q1 (N = 2273, < 2 days); Q2 (N = 3506, 2–4 days); Q3 (N = 2620, 5–9 days); and Q4 (N = 3213, ≥ 10 days). Both aORs for 28-day mortality and in-hospital mortality showed an increasing tendency with longer hospital LOS before RRS activation (Fig. 2).

### Subgroup analysis

**Fig. 3:**
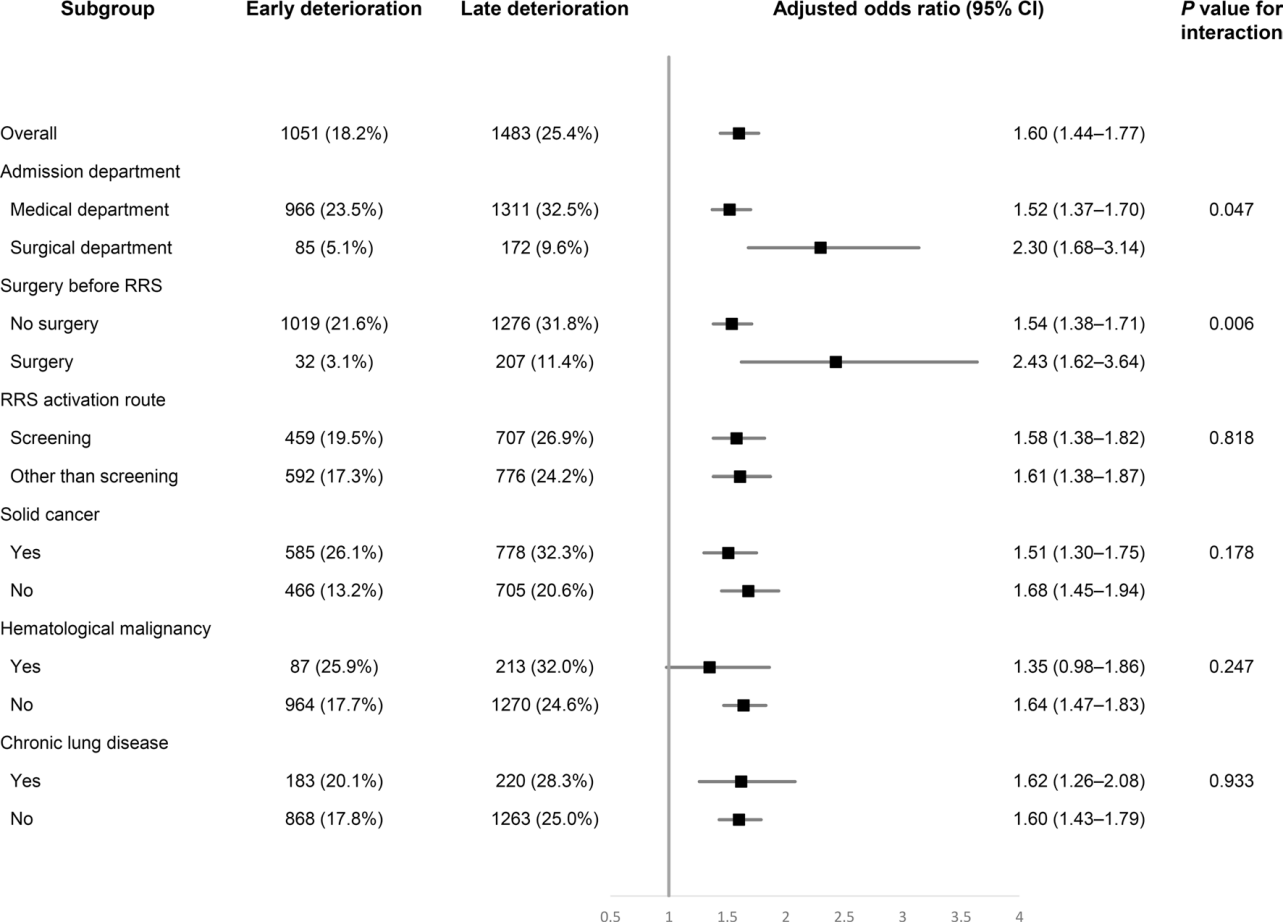
Forest plot for predefined subgroup analysis

Prespecified subgroup and interaction-term analyses were performed to investigate possible effect modifiers (Fig. 3). There was a significant interaction between the department of admission (medical vs. surgical) and the effects of hospital LOS on 28-day mortality (*P* = 0.047 for interaction). The negative impact of longer hospital LOS before RRS activation was stronger for patients admitted to the surgical department. A similar interaction was also observed when comparing patients who underwent a surgical operation and those who did not (*P* = 0.006 for interaction). For other subgroup analyses, there were no significant between-group differences in the primary outcome.

## Discussion

In this study, we found that longer hospital LOS before RRS activation was associated with worse clinical outcomes. Patients who stayed ≥ 5 days before RRS activation had higher 28-day and in-hospital mortality rates and were more likely to stay longer in the hospital after RRS activation compared with those who stayed < 5 days. This finding was robust after adjusting for variables reflecting the severity of illness at the time of RRS activation and even after propensity score-matched analysis.

The effectiveness of RRS has been studied extensively in the past two decades. Although early studies including cluster randomized trials failed to show significant reduction in mortality [13, 14], many other studies, such as before and after studies, have consistently shown positive results, as demonstrated in recent systematic review and meta-analysis studies [2, 15]. However, in real-world practice, when implementing RRS, it is difficult to predict clinical outcomes of individual patients for whom RRS activated because a wide range of patients with various comorbidities are being reviewed by the RRS team.

Certain alarming vital signs or laboratory test abnormalities are usually used as screening tools for RRS activation [16]. However, when two different patients with similar vital signs are reviewed by the RRS team, the expected outcomes may differ according to the patients’ current illness and comorbidities. As the volume of cases reviewed by the RRS team increases, it is important to be able to predict the clinical outcome to improve the cost-effectiveness and optimize resource use. This also relates to the decision about which patients should be admitted to the ICU when available beds and resources are limited. Patients who have a higher probability of recovery are usually given a higher priority for ICU admission [17]. However, it is difficult for RRS staff members to review the functional status and detailed medical history of patients and assess the likelihood of recovery in a short time.

In this regard, attention has been focused on efforts to identify predictors of clinical outcomes for patients for whom RRS is activated. A prospective observational study reported that assessment of frailty would be helpful for predicting the clinical trajectory of patients [18]. We hypothesized that hospital LOS before RRS activation may be a useful and simple predictor of clinical outcome after considering that severe frailty is usually associated with longer LOS [5]. In two previous single-center studies that evaluated the effect of LOS before RRS activation on clinical outcomes, the late deterioration group (≥ 7 days) had more than twice the in-hospital mortality rate than the early deterioration group (< 2 days) [19, 20]. However, those studies did not fully adjust for between-group differences in their analyses.

A study using the nationwide multicenter registry in the USA, which included about 280,000 patients, demonstrated that hours since admission before RRS activation was the second most important factor, after systolic blood pressure, in predicting in-hospital mortality [4]. However, a limitation of that study was that patients’ underlying comorbidities were categorized too simply as either medical or surgical and either cardiac or noncardiac. A detailed history of underlying comorbidities is a critical factor affecting the outcome, as shown in a recent study that reported an in-hospital mortality rate of > 40% in patients with hematological malignancy for whom RRS was activated [21].

In this study, we found that time since admission before RRS activation was an independent significant predictor of clinical outcome. A longer LOS before RRS activation itself may suggest ineffectiveness of the initial treatment and reflect the severity of the illness that caused the patient to be admitted. Therefore, among the patients with long hospital LOS at the time of RRS activation, invasive treatment, such as mechanical ventilation, may be deemed as futile in a certain proportion of patients. This is reflected by our finding that more patients in the late deterioration group had discussion with RRS staff members regarding the DNR order. Although attending physicians have a principal role in communicating with patients and their family members, the intervention of a third party, the RRS team, may improve end-of-life care planning by avoiding unnecessary or futile invasive treatment [22, 23].

The association between longer hospital LOS and worse clinical outcomes may indicate that medical problems acquired in the hospital setting are usually more serious, particularly for infectious complications [24]. This is because patients with hospital-acquired infection are at higher risk of infection with multidrug resistant pathogens. In our subgroup analysis, it was noted that the negative effects of longer hospital LOS on the clinical outcomes were more significant in patients who were admitted to surgical department or underwent a surgical operation. Given that postoperative wound infection or pneumonia are common problems leading to delay in discharge in surgical patients, these findings may be related to postoperative in-hospital infections due to difficult-to-treat pathogens [25].

Our study has several limitations. First, because of its retrospective observational design, we cannot exclude the possible effects of other unmeasured confounding factors. However, we found consistent results for the main analysis, propensity score-matched analysis, and several sensitivity analyses, which supports the robustness of our results. Second, we could not match every variable completely in our propensity score-matched analysis. Especially, the standardized difference between matched groups in the proportion of patients who underwent a surgical operation before RRS activation was 11.1%. Thus, we double adjusted the confounding variables to minimize the confounding effects [11]. Furthermore, we performed a subgroup analysis according to whether the patient received a surgery. Third, a causal relationship cannot be inferred between hospital LOS before RRS activation and later clinical outcomes. Despite these limitations, we believe that hospital LOS at the time of RRS activation may provide a simple and reliable prognostic information on future outcomes.

## Conclusions

In conclusion, among patients for whom RRS was activated for unexpected clinical deterioration during hospitalization, those who stayed longer in the hospital before RRS activation had a higher mortality rate than those who stayed a shorter time. For improving resource allocation without undermining the probability of recovery, a careful review of the reversibility of the patients should be performed in patients with a long hospital LOS at the time of RRS activation.

## Supplementary Information

**Additional file 1: Appendix S1.** RRS activation criteria of each center. **Appendix S2.** Supplemental methods. **Table S1.** Characteristics and outcomes of patients excluded in main analysis. **Table S2.** Information about situation when RRS was activated. **Table S3.** Full adjusted model for predicting 28-day mortality. **Table S4.** Characteristics of the propensity score-matched patients. **Table S5.** Outcome analysis with including all patients, not excluding those for whom RRS was activated after 1 month after admission. **Table S6.** Outcome analysis with patients without missing data. **Table S7.** Outcome analysis after excluding patients in whom discussion occurred regarding do-not-resuscitate orders. **Table S8.** Outcome analysis with handling hospital LOS before RRS activation as a continuous variable. **Table S9.** Outcome analysis based on categorizing patients into four quartiles according to LOS before RRS activation. **Figure S1.** Distribution of hospital length of stay before rapid response system activation. **Figure S2.** Propensity score distribution

## Data Availability

The datasets used and/or analyzed during the current study are available from the corresponding author on reasonable request.

## Abbreviations

CI: Confidence intervals
DNR: Do-not-resuscitate
IRR: Incident rate ratio
ICU: Intensive care unit
IQR: Interquartile range
LOS: Length of stay
MEWS: Modified early weaning score
NEWS2: National early warning score 2
OR: Odds ratio
Q: Quartile
RRS: Rapid response system
