# Supplementary material for: The association between hospital length of stay before rapid response system activation and clinical outcomes: a retrospective multicenter cohort study
Source: Respir Res. 2021 Feb 18;22:60. doi: 10.1186/s12931-021-01660-9 (PMC7891162; doi:10.1186/s12931-021-01660-9)

**Additional file 1**

**The association between hospital length of stay before rapid response system activation and clinical outcomes: a retrospective multicenter cohort study**

Jimyung Park^1*^, Yeon Joo Lee^2*^, Sang-Bum Hong^3^, Kyeongman Jeon^4,5^, Jae Young Moon^6^, Jung Soo Kim^7^, Byung Ju Kang^8^, Jong-Joon Ahn^8^, Dong-Hyun Lee^9^, Jisoo Park^10^, [Jae Hwa Cho](http://www.ncbi.nlm.nih.gov.ssl.libproxy.amc.seoul.kr:8000/pubmed/?term=Cho%20JH%5BAuthor%5D&cauthor=true&cauthor_uid=31795626)^11^, Sang-Min Lee^1^

^1^Division of Pulmonary and Critical Care Medicine, Department of Internal Medicine, Seoul National University Hospital, ^2^Division of Pulmonary and Critical Care Medicine, Department of Internal Medicine, Seoul National University Bundang Hospital, ^3^Department of Pulmonary and Critical Care Medicine, Asan Medical Center, University of Ulsan College of Medicine, ^4^Department of Critical Care Medicine, Samsung Medical Center, Sungkyunkwan University School of Medicine, ^5^Division of Pulmonary and Critical Care Medicine, Department of Medicine, Samsung Medical Center, Sungkyunkwan University School of Medicine, ^6^Division of Pulmonary and Critical Care Medicine, Department of Internal Medicine, Chungnam National University Sejong Hospital, Chungnam National University College of Medicine, ^7^Division of Pulmonary and Critical Care Medicine, Department of Internal Medicine, Inha University Hospital, Inha University School of Medicine, ^8^Department of Internal Medicine, Ulsan University Hospital, University of Ulsan College of Medicine, ^9^Division of Pulmonary and Critical Care Medicine, Department of Internal Medicine, Dong-A University College of Medicine, ^10^Division of Pulmonology, Department of Internal Medicine, CHA University, CHA Bundang Medical Center, ^11^Division of Pulmonology, Department of Internal Medicine, Gangnam Severance Hospital, Yonsei University College of Medicine

^*^ These authors contributed equally to this work

Corresponding author: Sang-Min Lee, M.D.

Division of Pulmonary and Critical Care Medicine, Department of Internal Medicine, Seoul National University College of Medicine, 101, Daehak-ro, Jongno-gu, Seoul, 03080, Republic of Korea, E-mail: [sangmin2@snu.ac.kr](mailto:sangmin2@snu.ac.kr)

**Table of contents**

**1. Appendix S1**

RRS activation criteria of each center

**2. Appendix S2**

Supplemental methods

**3. Table S1**

Characteristics and outcomes of patients excluded in main analysis

**4. Table S2**

Information about situation when RRS was activated

**5. Table S3**

Full adjusted model for predicting 28-day mortality

**6. Table S4**

Characteristics of the propensity score-matched patients

**7. Table S5**

Outcome analysis with including all patients, not excluding those for whom RRS was activated after 1 month after admission

**8. Table S6**

Outcome analysis with patients without missing data

**9. Table S7**

Outcome analysis after excluding patients in whom discussion occurred regarding do-not-resuscitate orders

**10. Table S8**

Outcome analysis with handling hospital LOS before RRS activation as a continuous variable

**11. Table S9**

Outcome analysis based on categorizing patients into four quartiles according to LOS before RRS activation

**12. Figure S1**

Distribution of hospital length of stay before rapid response system activation

**13. Figure S2**

Propensity score distribution

**Appendix S1. RRS activation criteria of each center**

1. Seoul National University Hospital

A. Calling criteria

- Respiratory: respiratory rate ≤8 or ≥28, SpO_2_ ≤90% for 5 minutes, dyspnea of sudden onset

- Cardiovascular: heart rate ≤40 or ≥130, systolic blood pressure ≤80 or ≥200, systolic blood pressure 80–90 with symptom, chest pain not responsive to sublingual nitroglycerin

- Neurologic: altered consciousness of sudden onset, sudden paralysis of face or extremities, new onset seizure, prolonged agitation (≥10 minutes) not fully explained by medical conditions

- Others: color change of peripheral extremities, subjective judgement of attending physician or nurse

B. Screening criteria

- Heart rate: <41, >129

- Respiratory rate: <9, >27

- Systolic blood pressure: <81, >199

- SpO_2_: <90%

2. Seoul National University Bundang Hospital

- Systolic blood pressure: <90

- Heart rate: <50, >140

- Respiratory rate: <10, >30

- Body temperature: <36°C, >39°C

- SpO_2_: <90%

- ABGA: pH <7.25, PaCO_2_ >50, PaO_2_ <55

- Lactate >4 mmol/L

- tCO_2_ <15 mmol/L

3. Asan Medical Center

A. Calling criteria

- Airway: threatened, stridor

- Breathing: respiratory rate <6 or >30, SpO_2_ <90% on Venturi 40% or O_2_ 6 L/min

- Circulation: heart rate <40 or >140, systolic blood pressure <90

- Neurology: sudden mental change, seizure

- Others: bedside nurse's concern about overall deterioration

B. Screening criteria

- Systolic blood pressure <86

- Sudden mental change or unexplained agitation

- Applying O_2_ >9 L/min, or FiO_2_ >35%

- Respiratory rate >27 or <8

- Unexplained heart rate >140 or <40

- Unexplained severe metabolic acidosis: pH <7.3, lactate >2 mmol/L, tCO_2_ <12 mmol/L

- PaCO_2_ >50 or PaO_2_ ≤55

- Bedside nurse concern about overall deterioration

4. Samsung Medical Center

A. Calling criteria

- Respiratory: respiratory rate ≥30, SpO_2_ <85% for 5 minutes, pH <7.3 & PaCO_2_ >50, stridor, use of accessary respiratory muscles

- Cardiovascular: systolic blood pressure <85, heart rate >130, acute chest pain, symptomatic arrhythmia

- Neurologic: altered mental status of sudden onset, unexplained agitation, seizure

- Others: bedside nurse concern about overall deterioration

B. Screening criteria

- MEWS (modified early warning score) ≥7

5. Chungnam National University Hospital

A. Calling criteria

- Airway: airway obstruction sign, stridor

- Breathing: respiratory rate <8 or >30, pH <7.3 & PaCO_2_ >60, SpO_2_ <90% on facial mask or high flow nasal cannula

- Circulation: heart rate <40 or >140, systolic blood pressure <90, lactate >2 mmol/L

- Neurology: sudden mental change or unexplained agitation, seizure

- Nurse’s concern about overall deterioration

B. Screening criteria

- based on NEWS (national early warning score)

6. Inha University Hospital

A. Calling criteria

- Breathing: respiratory rate ≤8 or ≥30, SpO_2_ <90% for 5 minutes on O_2_ >5 L/min, PaCO_2_ >50

- Circulation: systolic blood pressure <90, heart rate <40 or >130, acute chest pain

- Neurology: sudden mental change, seizure

- Others: bedside nurse’s concern about overall deterioration

B. Screening criteria

- Breathing: respiratory rate ≤8 or ≥25, SpO_2_ ≤90%, PaO_2_ ≤55, PaCO_2_ ≥50, pH ≤7.3, lactate ≥2 mmol/L, tCO_2_ ≤12 mmol/L

- Circulation: systolic blood pressure ≤90, heart rate ≤40 or ≥130, acute chest pain

- Neurology: sudden mental change, seizure

- 48 hours after discharge from ICU

- High risk surgical patients

7. Ulsan University Hospital

A. Calling criteria

- Airway: threatened airway, stridor, wheezing sound

- Breathing: respiratory rate ≤6 or ≥30, SpO_2_ <90% on O_2_ 6 L/min or venturi mask 40%

- Circulation: heart rate <40 or ≥140, systolic blood pressure <90

- Neurology: sudden mental change, seizure

- Others: bedside nurse’s concern about overall deterioration

B. Screening criteria

- Airway: threatened airway, stridor, wheezing sound

- Breathing: respiratory rate ≤6 or ≥30, SpO_2_ <90%, oxygen demand (over nasal prong 6 L/min or venturi mask 40%)

- Circulation: heart rate <40 or ≥140, systolic blood pressure ≤85

- Neurology: sudden mental change, seizure

- Others: pH ≤7.33, HCO_3_ ≤15 mmol/L, lactate ≥2 mmol/L, potassium ≥6 mmol/L, tCO_2_ ≤15 mmol/L, glucose ≤50 mg/dL, ICU discharge patients

8. Dong-A University Hospital

- respiratory rate >28 or <8

- heart rate >140 or <40

- systolic blood pressure <90

- ABGA: pH <7.3 or >7.6, HCO_3_ <14 mmol/L, PaCO_2_ >50, PaO_2_ <55

- Lactate >30 mg/dL, tCO_2_ <15 mmol/L, potassium >6 or <3 mmol/L

- SpO_2_ <90%, O_2_ >8 L/min

9. CHA Bundang Medical Center

- mean blood pressure <60 or systolic blood pressure <90

- applying O_2_ >9 L/min or venturi mask >35%

- respiratory rate >25 or <8

- heart rate >140 or <40

- glucose ≤50 mg/dL

- unexplained severe metabolic acidosis: pH<7.3 or lactate >2 mmol/L or tCO_2_ <16 mmol/L

- PaO_2_ ≤55 or PaCO_2_ >50

- sudden mental change or unexplained agitation, unexplained seizures

- chest pain, upper airway obstruction sign like stridor

- bedside nurse concern about overall deterioration

**Appendix S2. Supplemental methods**

Data collection

The extracted data included demographic characteristics such as sex, age, height, weight, the date of admission to and discharge from the hospital, the main reason for hospitalization, and underlying comorbidities. In addition, detailed information regarding the situation when RRS was activated was collected as follows: date, time, and location of RRS activation; whether the patient was in the postoperative state when RRS was activated; assessment of the patient made by the RRS team; and interventions carried out by the RRS team. We also checked whether discussion of a do-not-resuscitate (DNR) order was made after RRS activation. Whether the patient was admitted to the ICU after RRS activation was reviewed and, in such cases, the date of discharge from the ICU was used to calculate the LOS in the ICU. To evaluate outcomes related to survival (28-day mortality and in-hospital mortality), we analyzed whether the patients included had died or survived within the 28 days after RRS activation and whether they were discharged from the hospital alive.

The severity of illness at the time of RRS activation was evaluated using information on vital signs, level of consciousness, and whether the patient needed supplemental oxygen therapy. Using these data, the modified early weaning score (MEWS) and the national early warning score 2 (NEWS2) were calculated for each patient. The following data from laboratory test results were gathered when these tests had been performed: arterial blood gas analysis; complete blood count (white blood cell, hemoglobin, and platelet counts); prothrombin time; serum chemistry to evaluate hepatic and renal function; and C-reactive protein concentration. If relevant laboratory test results were available, the sequential organ failure assessment (SOFA) score was calculated.

Statistical analysis

The descriptive data for continuous variables are summarized as medians with interquartile ranges (IQRs) because of their nonnormal distribution. For categorical variables, the values are reported as frequencies with proportions. Mortality outcomes and rates of admission to the ICU were compared between the early and late deterioration groups using a multivariable logistic regression and adjusted odds ratios (aORs) with 95% confidence intervals (CIs) were calculated. After performing repeated univariable logistic regressions, clinically relevant variables showing significant associations with mortality outcomes were selected and adjusted for in subsequent multivariable regressions as follows: age; department of admission (medical vs. surgical); whether patients were in the postoperative state; comorbidities such as solid cancer, hematological malignancy, chronic lung disease, hepatobiliary disease, or cerebrovascular disease; and whether discussion on DNR was made after RRS activation.

To adjust for the severity of illness at the time of RRS activation in outcome analysis, we evaluated the associations of MEWS and NEWS2 with mortality outcomes. In the multivariable model, we decided to use NEWS2 because the 95% CI was narrower for NEWS2 than for MEWS. We used only NEWS2, but did not adjust for other variables related to illness severity such as vital signs because we considered that this could lead to a collinearity issue. We did not consider laboratory test results as variables to adjust for because not every patient had the available data. The performance of the multivariable logistic regression model was assessed using C-statistics. Outcomes related to LOS (hospital LOS after RRS activation and LOS in the ICU for patients admitted to the ICU) were analyzed using a negative binomial regression to calculate the adjusted incidence rate ratios (aIRRs), after adjusting for the same variables as described above.

Propensity score-matched analysis

We performed propensity score-matched analysis to reduce the effects of possible confounding factors as much as possible. The propensity score reflected each patient’s probability of belonging to the late deterioration group rather than the early deterioration group and was estimated using a logistic regression model with every available baseline patient characteristic. We then conducted 1:1 optimal matching without replacement based on the propensity score within a specified caliper ≤0.05. Any imbalance between matched groups (early vs. late deterioration) was assessed using the standardized differences, and a standardized difference <10% was considered to indicate a well-matched state.

Using matched samples, we calculated ORs to assess the associations between binary outcomes and LOS before RRS activation using a conditional logistic regression model for matched data. Both unadjusted and adjusted analyses were performed to minimize any remaining imbalance after matching. For continuous outcomes, the Wilcoxon signed-rank test was used to compare between matched samples.

**Table S1. Characteristics and outcomes of patients excluded in main analysis**

| Variables | Patients  included  (N = 11,612) | Patients  excluded^1^  (N = 1,191) | *P* value |
| --- | --- | --- | --- |
| Age (yr) | 68 (57–76) | 65 (54–75) | <0.001 |
| Male sex | 6,785 (58.4%) | 723 (60.7%) | 0.129 |
| Body mass index (kg/m^2^) | 22.2 (19.6–25.0) | 21.2 (18.6–24.1) | <0.001 |
| Admission to medical department | 8,160 (70.3%) | 843 (70.8%) | 0.714 |
| Surgical operation  before RRS activation | 2,867 (24.7%) | 518 (43.5%) | <0.001 |
| Comorbidity  Solid cancer  Hematological malignancy  Cardiovascular disease  Diabetes mellitus  Chronic lung disease  Hepatobiliary disease  Chronic kidney disease  Cerebrovascular disease  Organ transplantation | 4,646 (40.0%)  1,002 (8.6%)  2,715 (23.4%)  3,132 (27.0%)  1,687 (14.5%)  1,297 (11.2%)  1,251 (10.8%)  1,329 (11.5%)  356 (3.1%) | 342 (28.7%)  215 (18.1%)  286 (24.0%)  338 (28.4%)  141 (11.8%)  142 (11.9%)  130 (10.9%)  186 (15.6%)  110 (9.2%) | <0.001  <0.001  0.624  0.298  0.012  0.433  0.881  <0.001  <0.001 |
| Hospital LOS before  RRS activation (days) | 5 (2–10) | 49 (38–73) | <0.001 |
| Illness severity at RRS activation  MEWS  NEWS2 | 4 (2–5)  7 (5–10) | 5 (3–6)  8 (6–11) | <0.001  <0.001 |
| Discussion on do-not-resuscitate order | 1,226 (10.6%) | 133 (11.2%) | 0.516 |
| 28-day mortality | 2,534 (21.8%) | 370 (31.1%) | <0.001 |
| In-hospital mortality | 2,967 (25.6%) | 499 (42.5%) | <0.001 |
| Admission to intensive care unit | 3,385 (29.2%) | 438 (36.8%) | <0.001 |

RRS, Rapid response system; LOS, Length of stay; MEWS, Modified early weaning score; NEWS2, National early warning score 2

^1^ These patients were excluded because they had been staying in the hospital more than 1 month at the time of RRS activation, according to the inclusion/exclusion criteria of this study. This is reflected by very long hospital LOS (median 49 days) before RRS activation.

**Table S2. Information about situation when RRS was activated**

| Variables | Total  patients  (N = 11,612) | Early  deterioration  (N = 5,779) | Late  deterioration  (N = 5,833) |
| --- | --- | --- | --- |
| Location  General ward  Non-general ward^1^ | 11,394 (98.1%)  218 (1.9%) | 5,630 (97.4%)  149 (2.6%) | 5,764 (98.8%)  69 (1.2%) |
| Route of activation  Screening criteria  Call from doctors  Call from nurses  Cardiopulmonary arrest  Others^2^ | 6,630 (57.1%)  2,909 (25.1%)  1,340 (11.5%)  417 (3.6%)  316 (2.7%) | 3,426 (59.3%)  1,430 (24.7%)  654 (11.3%)  179 (3.1%)  90 (1.6%) | 3,204 (54.9%)  1,479 (25.3%)  686 (11.8%)  238 (4.1%)  226 (3.9%) |
| Assessment made by RRS team  Respiratory distress  Sepsis & Septic shock  Arrhythmia  Hypovolemic shock  Altered mentality  Metabolic acidosis  Cardiac arrest  Cardiogenic shock  Others^3^ | 4,073 (35.1%)  910 (7.8%)  706 (6.1%)  621 (5.3%)  508 (4.4%)  479 (4.1%)  337 (2.9%)  76 (0.7%)  3,902 (33.6%) | 1,924 (33.3%)  462 (8.0%)  349 (6.0%)  312 (5.4%)  224 (3.9%)  265 (4.6%)  138 (2.4%)  43 (0.7%)  2,062 (35.7%) | 2,149 (36.8%)  448 (7.7%)  357 (6.1%)  309 (5.3%)  284 (4.9%)  214 (3.7%)  199 (3.4%)  33 (0.6%)  1,840 (31.5%) |

RRS, Rapid response system

^1^ Non-general ward includes laboratory unit, dialysis unit, operating room, hospital lobby floor, etc.

^2^ Others include calls from hospital staff members other than doctors and nurses, and coincidental case detections by RRS team members themselves.

^3^ A variety of conditions are included for other assessments made by RRS team, such as obstructive shock due to pulmonary embolism or cardiac tamponade, anaphylactic shock, excessively high blood pressure from various causes, transient instability of vital signs related to medical procedures, RRS calls for close monitoring during transfer of the patients, and so on.

**Table S3. Full adjusted model for predicting 28-day mortality**

| Variables | Unadjusted  odds ratio | *P* value | Adjusted  odds ratio | *P* value |
| --- | --- | --- | --- | --- |
| Age  (per year) | 1.01  (1.00–1.01) | 0.001 | 1.01  (1.00–1.01) | <0.001 |
| Admission to medical department  (vs. Surgical department) | 4.81  (4.20–5.51) | <0.001 | 2.44  (2.06–2.90) | <0.001 |
| Surgery before RRS activation  (vs. No surgery) | 0.26  (0.22–0.29) | <0.001 | 0.56  (0.46–0.67) | <0.001 |
| History of solid cancer  (vs. No history) | 2.05  (1.88–2.25) | <0.001 | 1.87  (1.67–2.08) | <0.001 |
| History of hematological malignancy  (vs. No history) | 1.60  (1.39–1.85) | <0.001 | 1.51  (1.28–1.79) | <0.001 |
| History of chronic lung disease  (vs. No history) | 1.15  (1.02–1.30) | 0.026 | 1.05  (0.91–1.21) | 0.504 |
| History of hepatobiliary disease  (vs. No history) | 1.34  (1.17–1.53) | <0.001 | 1.35  (1.16–1.57) | <0.001 |
| History of cerebrovascular disease  (vs. No history) | 0.65  (0.56–0.76) | <0.001 | 0.62  (0.52–0.74) | <0.001 |
| Discussion on do-not-resuscitate order  (vs. No discussion) | 10.22  (8.98–11.63) | <0.001 | 6.08  (5.30–6.99) | <0.001 |
| NEWS2  (per 1 point) | 1.20  (1.18–1.21) | <0.001 | 1.15  (1.14–1.17) | <0.001 |
| Late deterioration group  (vs. Early deterioration group) | 1.53  (1.40–1.68) | <0.001 | 1.60  (1.44–1.77) | <0.001 |

RRS, Rapid response system; NEWS2, National early warning score 2

**Table S4. Characteristics of the propensity score-matched patients**

| Variables | Early  deterioration  (N = 4,454) | Late  deterioration  (N = 4,454) | Standardized difference (%) |
| --- | --- | --- | --- |
| Age (yr) | 68 (57–77) | 68 (57–77) | 0.6 |
| Male sex | 2,630 (59.1%) | 2,669 (59.9%) | 1.8 |
| Body mass index (kg/m^2^) | 22.2 (19.7–24.9) | 22.2 (19.6–25.0) | 1.1 |
| Admission to medical department | 3,319 (74.5%) | 3,184 (71.5%) | 6.8 |
| Surgical operation before RRS activation | 865 (19.4%) | 1,068 (24.0%) | 11.1 |
| History of solid cancer | 1,855 (41.7%) | 1,860 (41.8%) | 0.2 |
| History of hematological malignancy | 317 (7.1%) | 437 (9.8%) | 9.7 |
| History of cardiovascular disease | 1,049 (23.6%) | 1,070 (24.0%) | 1.1 |
| History of diabetes mellitus | 1,192 (26.8%) | 1,217 (27.3%) | 1.3 |
| History of chronic lung disease | 691 (15.5%) | 639 (14.4%) | 3.3 |
| History of hepatobiliary disease | 499 (11.2%) | 494 (11.1%) | 0.4 |
| History of chronic kidney disease | 467 (10.5%) | 471 (10.6%) | 0.3 |
| History of cerebrovascular disease | 485 (10.9%) | 519 (11.7%) | 2.4 |
| History of organ transplantation | 111 (2.5%) | 115 (2.6%) | 0.6 |
| RRS activation in general ward | 4,394 (98.7%) | 4,406 (98.9%) | 2.5 |
| RRS activation by screening criteria | 2,621 (58.9%) | 2,572 (57.8%) | 2.5 |
| Mean blood pressure | 83 (68–98) | 83 (67–99) | 0.4 |
| Herat rate | 103 (85–122) | 104 (87–122) | 3.2 |
| Respiratory rate | 22 (19–28) | 22 (19–28) | 0.1 |
| Body temperature | 36.9 (36.5–37.6) | 36.9 (36.5–37.6) | 0.5 |
| Alert mental status | 3,232 (72.6%) | 3,114 (69.9%) | 5.9 |
| Supplemental oxygen therapy | 2,761 (62.0) | 2,793 (62.7%) | 1.5 |
| MEWS | 4 (2–5) | 4 (2–5) | 4.7 |
| NEWS2 | 7 (5–10) | 7 (5–10) | 3.7 |
| Tracheal intubation by RRS | 571 (12.8%) | 589 (13.2%) | 1.2 |
| Initiation of mechanical ventilation | 536 (12.0%) | 556 (12.5%) | 1.4 |
| Application of high flow nasal cannula | 382 (8.6%) | 364 (8.2%) | 1.5 |
| Initiation of noninvasive ventilation | 58 (1.3%) | 60 (1.4%) | 0.4 |
| Renal replacement therapy | 206 (4.6%) | 201 (4.5%) | 0.5 |
| ACLS | 103 (2.3%) | 109 (2.5%) | 0.9 |
| ECLS | 16 (0.4%) | 16 (0.4%) | 0.0 |
| Central venous catheterization | 332 (7.5%) | 320 (7.2%) | 1.0 |
| Initiation of new antimicrobial therapy | 290 (6.5%) | 311 (7.0%) | 1.9 |
| Starting vasopressor/inotrope | 550 (12.4%) | 562 (12.6%) | 0.8 |
| Transfusion | 246 (5.5%) | 258 (5.8%) | 1.2 |
| Discussion on do-not-resuscitate order | 485 (10.9%) | 528 (11.9%) | 3.0 |

RRS, Rapid response system; MEWS, Modified early weaning score); NEWS2, National early warning score 2; ACLS, Advanced cardiac life support; ECLS, Extracorporeal life support

**Table S5. Outcome analysis with including all patients, not excluding those for whom RRS was activated after 1 month after admission**

| Variables | Adjusted OR or IRR^1^  (Late deterioration vs. Early deterioration) | *P* value |
| --- | --- | --- |
| Primary outcome |  |  |
| 28-day mortality | 1.64 (1.47–1.82) | <0.001 |
|  |  |  |
| Secondary outcome |  |  |
| In-hospital mortality | 1.84 (1.67–2.02) | <0.001 |
| ICU admission | 1.09 (1.00–1.19) | 0.044 |
| Hospital LOS after RRS activation | 1.45 (1.39–1.51) | <0.001 |
| LOS in ICU^2^ | 1.30 (1.22–1.40) | <0.001 |

LOS, Length of stay; RRS, Rapid response system; OR, Odds ratio; IRR, Incidence rate ratio; ICU, intensive care unit

^1^ Multivariable logistic regression and negative binomial regression were performed with adjusting for following confounding variables: age, department of admission (medical vs. surgical), whether patients were in postoperative state, comorbidities of solid cancer, hematological malignancy, chronic lung disease, hepatobiliary disease, or cerebrovascular disease, whether DNR (do-not-resuscitate) discussion was made after RRS activation, and NEWS2 (national early warning score 2).

^2^ Analysis on LOS in ICU included only patients who were admitted to the ICU.

**Table S6. Outcome analysis with patients without missing data**

| Variables | Adjusted OR or IRR^1^ | | *P* value |
| --- | --- | --- | --- |
|  | Early  deterioration | Late  deterioration |  |
| Primary outcome |  |  |  |
| 28-day mortality | 1.00 | 1.61 (1.44–1.81) | <0.001 |
|  |  |  |  |
| Secondary outcome |  |  |  |
| In-hospital mortality | 1.00 | 1.70 (1.53–1.89) | <0.001 |
| ICU admission | 1.00 | 1.14 (1.04–1.24) | 0.005 |
| Hospital LOS after RRS activation | 1.00 | 1.34 (1.29–1.39) | <0.001 |
| LOS in ICU^2^ | 1.00 | 1.21 (1.12–1.30) | <0.001 |

LOS, Length of stay; RRS, Rapid response system; OR, Odds ratio; IRR, Incidence rate ratio; ICU, intensive care unit

^1^ Multivariable logistic regression and negative binomial regression were performed with adjusting for following confounding variables: age, department of admission (medical vs. surgical), whether patients were in postoperative state, comorbidities of solid cancer, hematological malignancy, chronic lung disease, hepatobiliary disease, or cerebrovascular disease, whether DNR (do-not-resuscitate) discussion was made after RRS activation, and NEWS2 (national early warning score 2).

^2^ Analysis on LOS in ICU included only patients who were admitted to the ICU.

**Table S7. Outcome analysis after excluding patients in whom discussion occurred regarding do-not-resuscitate orders**

| Variables | Adjusted OR or IRR^1^ | | *P* value |
| --- | --- | --- | --- |
|  | Early  deterioration | Late  deterioration |  |
| Primary outcome |  |  |  |
| 28-day mortality | 1.00 | 1.58 (1.42–1.76) | <0.001 |
|  |  |  |  |
| Secondary outcome |  |  |  |
| In-hospital mortality | 1.00 | 1.69 (1.52–1.87) | <0.001 |
| ICU admission | 1.00 | 1.09 (0.99–1.19) | 0.073 |
| Hospital LOS after RRS activation | 1.00 | 1.29 (1.24–1.34) | <0.001 |
| LOS in ICU^2^ | 1.00 | 1.26 (1.17–1.35) | <0.001 |

LOS, Length of stay; RRS, Rapid response system; OR, Odds ratio; IRR, Incidence rate ratio; ICU, intensive care unit

^1^ Multivariable logistic regression and negative binomial regression were performed with adjusting for following confounding variables: age, department of admission (medical vs. surgical), whether patients were in postoperative state, comorbidities of solid cancer, hematological malignancy, chronic lung disease, hepatobiliary disease, or cerebrovascular disease, whether DNR (do-not-resuscitate) discussion was made after RRS activation, and NEWS2 (national early warning score 2).

^2^ Analysis on LOS in ICU included only patients who were admitted to the ICU.

**Table S8. Outcome analysis with handling hospital LOS before RRS activation as a continuous variable**

| Variables | Adjusted OR or IRR^1^  (per 1 day increase in hospital LOS) | *P* value |
| --- | --- | --- |
| Primary outcome |  |  |
| 28-day mortality | 1.033 (1.026–1.040) | <0.001 |
|  |  |  |
| Secondary outcome |  |  |
| In-hospital mortality | 1.039 (1.033–1.046) | <0.001 |
| ICU admission | 1.010 (1.004–1.016) | 0.001 |
| Hospital LOS after RRS activation | 1.021 (1.019–1.024) | <0.001 |
| LOS in ICU^2^ | 1.015 (1.009–1.019) | <0.001 |

LOS, Length of stay; RRS, Rapid response system; OR, Odds ratio; IRR, Incidence rate ratio; ICU, intensive care unit

^1^ Multivariable logistic regression and negative binomial regression were performed with adjusting for following confounding variables: age, department of admission (medical vs. surgical), whether patients were in postoperative state, comorbidities of solid cancer, hematological malignancy, chronic lung disease, hepatobiliary disease, or cerebrovascular disease, whether DNR (do-not-resuscitate) discussion was made after RRS activation, and NEWS2 (national early warning score 2).

^2^ Analysis on LOS in ICU included only patients who were admitted to the ICU.

**Table S9. Outcome analysis based on categorizing patients into four quartiles according to LOS before RRS activation**

| Variables | Value | Adjusted OR^1^ | *P* value^2^ |
| --- | --- | --- | --- |
| 28-day mortality | 2,534 (21.8%) |  |  |
| Q1 (N = 2,273): LOS < 2 days | 421 (18.5%) | 1.00 |  |
| Q2 (N = 3,506): 2 days ≤ LOS < 5 days | 630 (18.0%) | 1.24 (1.06–1.44) | 0.006 |
| Q3 (N = 2,620): 5 days ≤ LOS <10 days | 589 (22.5%) | 1.59 (1.36–1.87) | <0.001 |
| Q4 (N = 3,213): 10 days ≤ LOS ≤ 1 month | 894 (27.8%) | 2.00 (1.72–2.33) | <0.001 |
|  |  |  |  |
| In-hospital mortality | 2,967 (25.6%) |  |  |
| Q1 (N = 2,273): LOS < 2 days | 486 (21.4%) | 1.00 |  |
| Q2 (N = 3,506): 2 days ≤ LOS < 5 days | 723 (20.7%) | 1.22 (1.06–1.41) | 0.006 |
| Q3 (N = 2,620): 5 days ≤ LOS <10 days | 684 (26.1%) | 1.63 (1.40–1.90) | <0.001 |
| Q4 (N = 3,213): 10 days ≤ LOS ≤ 1 month | 1,074 (33.5%) | 2.17 (1.89–2.50) | <0.001 |

LOS, Length of stay; RRS, Rapid response system; OR, Odds ratio

^1^ Multivariable logistic regression was performed with adjusting for following confounding variables: age, department of admission (medical vs. surgical), whether patients were in postoperative state, comorbidities of solid cancer, hematological malignancy, chronic lung disease, hepatobiliary disease, or cerebrovascular disease, whether DNR (do-not-resuscitate) discussion was made after RRS activation, and NEWS2 (national early warning score 2).

^2^ *P* values are based on statistical comparisons with Q1 as a reference group.

**Figure S1.** Distribution of hospital length of stay before rapid response system activation


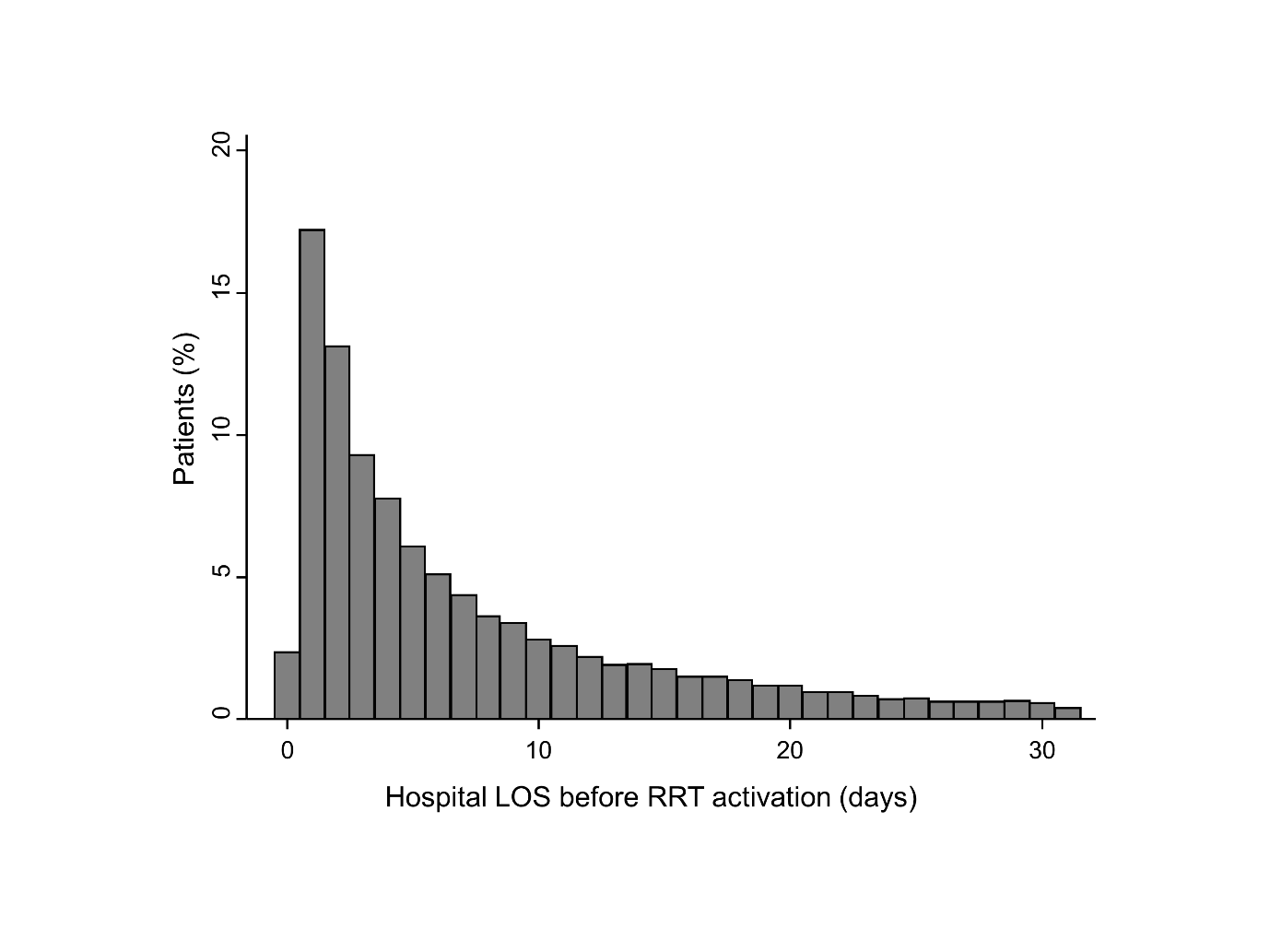


**Figure S2.** Propensity score distribution


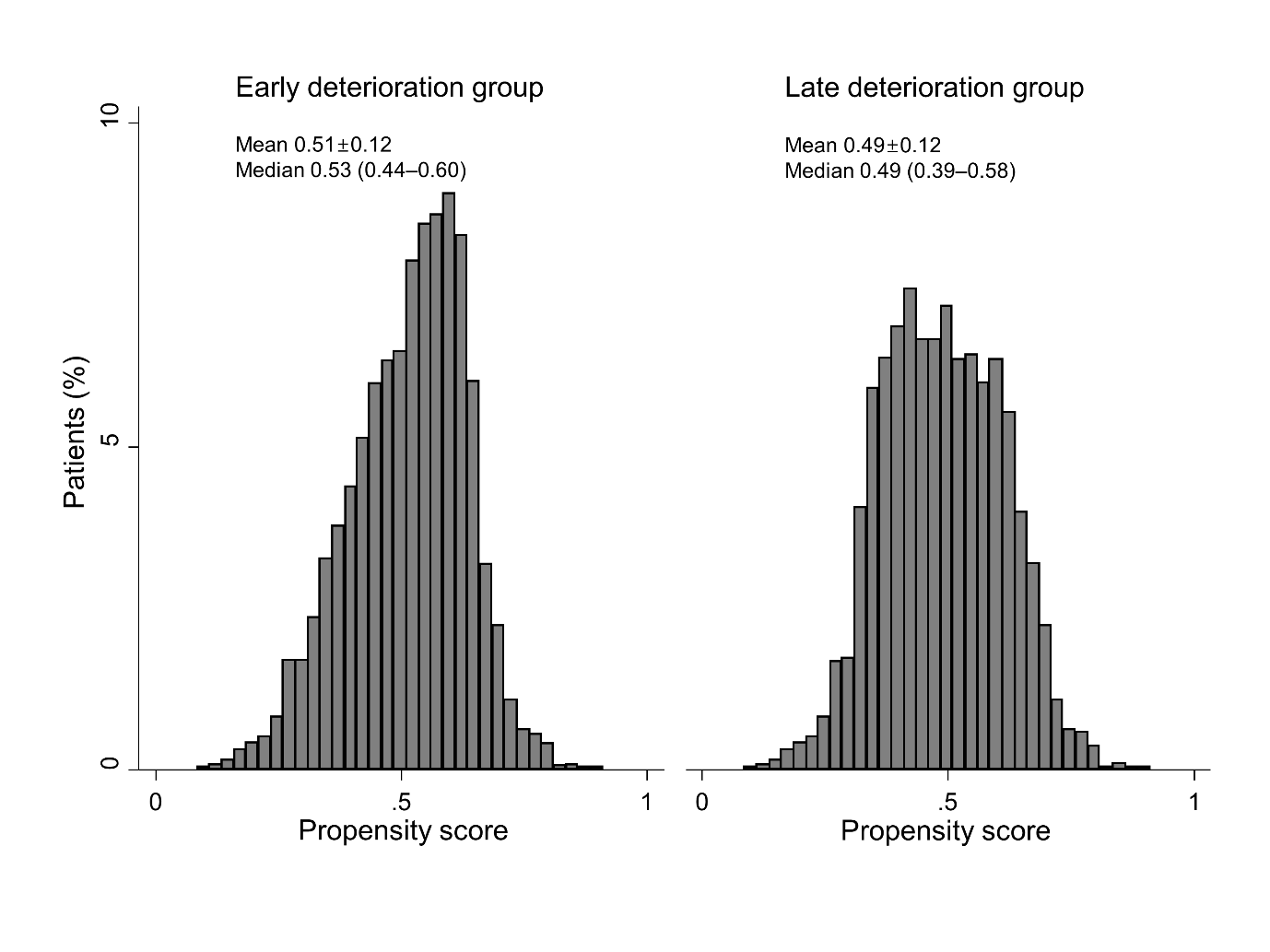

Supplement: Supplementary file 1 — Additional file 1: Appendix S1. RRS activation criteria of each center. Appendix S2. Supplemental methods. Table S1. Characteristics and outcomes of patients excluded in main analysis. Table S2. Information about situation when RRS was activated. Table S3. Full adjusted model for predicting 28-day mortality. Table S4. Characteristics of the propensity score-matched patients. Table S5. Outcome analysis with including all patients, not excluding those for whom RRS was activated after 1 month after admission. Table S6. Outcome analysis with patients without missing data. Table S7. Outcome analysis after excluding patients in whom discussion occurred regarding do-not-resuscitate orders. Table S8. Outcome analysis with handling hospital LOS before RRS activation as a continuous variable. Table S9. Outcome analysis based on categorizing patients into four quartiles according to LOS before RRS activation. Figure S1. Distribution of hospital length of stay before rapid response system activation. Figure S2. Propensity score distribution [file 12931_2021_1660_MOESM1_ESM.docx]
